# Blocking p38 Signaling Reduces the Activation of Pro-inflammatory Cytokines and the Phosphorylation of p38 in the Habenula and Reverses Depressive-Like Behaviors Induced by Neuroinflammation

**DOI:** 10.3389/fphar.2018.00511

**Published:** 2018-05-15

**Authors:** Ya-wei Zhao, Yu-qin Pan, Ming-ming Tang, Wen-juan Lin

**Affiliations:** ^1^Key Laboratory of Mental Health, Institute of Psychology, Chinese Academy of Sciences, Beijing, China; ^2^Department of Psychology, University of Chinese Academy of Sciences, Beijing, China

**Keywords:** depression, p38, neuroinflammation, habenula, fluoxetine, SB203580

## Abstract

Increasing evidence has demonstrated that neuroinflammation contributes to the development of depressive-like behaviors, in both animal models and human patients; however, the brain areas and signaling pathways involved are still elusive. Recent studies have suggested novel roles of the habenula in the onset of depression and other psychiatric disorders; however, there is no evidence for whether the habenula has a function in neuroinflammation-induced depression. Using an animal model of depression, which is induced by the repeated central administration of lipopolysaccharide (LPS), we examined whether cytokine expression and p38 signal activation in the habenula were involved in the depressive-like behaviors. Body weight, saccharin preference test, and tail suspension test were used to measure depressive-like behaviors. Immunohistochemistry, quantitative-polymerase chain reaction (q-PCR), and western blot were used to measure the expression of tumor necrosis factor-α (TNF-α), interleukin-10 (IL-10), and the phosphorylation of p38 in the habenula. The results showed that central LPS administration induced depressive-like behaviors, characterized by anhedonia in the saccharin preference test and increased immobility in the tail suspension test. Central LPS administration also significantly increased the p-p38 level in microglial cells and increased TNF-α expression in the habenula. Treatment with fluoxetine, a widely prescribed antidepressant, or SB203580, a p38-specific inhibitor, reversed the depressive-like behaviors, normalized the alterations in p-p38 and TNF-α levels and increased the levels of the anti-inflammatory cytokine IL-10 in the habenula. The present findings suggest that the habenula is involved in the pathophysiology of behavioral depression induced by neuroinflammation, and the p38 pathway may serve as a novel mechanism-based target for the treatment of inflammation-related depression.

## Introduction

Depression is a devastating mental disorder, characterized by depressed mood, anhedonia, and reduced energy (Phillips and Kupfer, 2013). The etiology of depression is complex and still not fully understood. Although recent therapeutic strategies have focused on the regulation of the monoamine transmitter system (Goldberg et al., 2014; Haase and Brown, 2015), increasing evidence suggests that immune activation plays important roles in triggering depression (Pedro et al., 2018). By expressing pro-inflammatory cytokines, the immune system can cause depressive-like behaviors and changes in the neuroendocrine and neurochemical system, a phenomenon called “the cytokine hypothesis of depression” (Jeon and Kim, 2016; Shariq et al., 2018). TNF-α, interleukin-1β (IL-1β), and interleukin-6 (IL-6) are the pro-inflammatory cytokines that have been most intensively studied in the field (Kim et al., 2016; Sordillo et al., 2016). TNF-α has long been thought to be involved in the etiology of depression (Himmerich et al., 2008; Liu et al., 2015) and is suggested to be a major and common risk factor for inflammation-associated depressive disorders, as its mRNA expression levels were elevated in models of depression induced by both chronic stress and repeated central LPS infusion (Guan et al., 2015). Anti-inflammatory cytokines can modulate the action of central pro-inflammatory cytokines (Dhabhar et al., 2009; Xu and Lin, 2014). It has been reported that varying expression levels of IL-10, a typical and potent anti-inflammatory cytokine, are associated with altered depressive-like behaviors (Mesquita et al., 2008), and central IL-10 administration was reported to reverse depressive-like behaviors induced by forced swim stress (Pan et al., 2013).

Immune system activation can induce the activation of numerous signaling pathways that cause behavioral changes, and the mitogen-activated protein kinases (MAPKs) pathway has received the most attention (Duric et al., 2010; Reus et al., 2014). MAPKs are members of a superfamily of serine/threonine protein kinases that play crucial roles in regulating the expression of genes involved in a wide variety of cellular processes (Kim and Choi, 2010; Lloberas et al., 2016; Saba-El-Leil et al., 2016). There are three major groups of MAPKs: the extracellular signal-related kinases 1/2 (ERK1/2), the c-Jun N-terminal kinases 1/2 (JNK1/2), and p38 MAPK. P38 MAPK, one of the best studied MAPKs, was found to influence a multitude of cellular events, such as cell growth and death, cell proliferation, differentiation, and inflammation and is a stress-activated kinase that plays an important role in the regulation of cytokine production in the immune system (Cuadrado and Nebreda, 2010; Li et al., 2015). Increasing evidence indicates that p38 MAPK may be part of an important signaling pathway that mediates behavioral changes in depression (Felger et al., 2011; Galeotti and Ghelardini, 2012; Baganz et al., 2015). Research showed that SB203580, a p38-specific inhibitor, can exert an anti-depressant effect by decreasing the immobility induced by a forced swim test (FST) (Felger et al., 2011). Lipopolysaccharide (LPS) is the major component of the outer membrane of Gram-negative bacteria, which can induce a strong response from animal immune systems. In response to LPS, the activation of p38 MAPK has been demonstrated to be an essential signaling mechanism governing the regulation of TNF-α expression in neutrophils and macrophages (Miller et al., 2013). However, whether the p38 MAPK pathway has a mediating role in neuroinflammation-related depression remains unclear.

The etiology of depression has been associated with many areas in the brain. Although the brain regions of the prefrontal cortex, amygdala, and hippocampus are important for stress- or inflammation-induced depression, many studies are increasingly suggesting a novel, circuit-based paradigm, through which the habenula may be causally involved in the onset of depression and other psychiatric illnesses (Boulos et al., 2017).

The habenula, a relay station connecting the forebrain and midbrain, is the hub for emotion regulation (Fakhoury, 2017; Fore et al., 2017). It is located bilaterally behind the thalamus, near the midline of birds and mammals and is divided into two parts, the medial habenula (MHb) and the lateral habenula (LHb) (Aizawa et al., 2011), which maintains close interactions with numerous brain regions, such as the limbic system and the basal ganglia (Beretta et al., 2012). It has been suggested that the habenula may play a role in aversive processing and may contribute to the generation of depression symptoms, such as anhedonia (Lawson et al., 2017). The first evidence indicating a role of the habenula in emotional disorders was discovered in monkeys. The habenula was found to be activated during the anticipation of aversive outcomes or during the failure to obtain a reward. The activation of the habenula leads to reduced motor behaviors (Matsumoto and Hikosaka, 2007). This discovery attracted attention to this brain region in the pathophysiology of depression. It was found that LHb lesions in depressed rats improved behavioral responses, decreased immobility time in the FST, and increased climbing behavior (Yang et al., 2008). Increased presynaptic activity onto LHb neurons contributes to the rodent learned helplessness model of depression (Li et al., 2011), and inhibiting LHb activation using the gamma-aminobutyric acid (GABA) agonist muscimol could exert anti-depressant effects in congenital helpless rats (Winter et al., 2011). However, little research has been performed to study the effect of immune activation in the habenula on depression. The only study discovered that acute or chronic restraint stress induced a strong elevation of interleukin-18 (IL-18) levels in the MHb (Sugama et al., 2002).

Thus, the purpose of the present study was to investigate whether p38 signals participate in neuroinflammation-induced depressive-like behaviors. We used repeated central LPS injection to induce depressive-like behaviors, which were tested by a series of behavioral tests. The levels of the cytokines TNF-α, IL-10, and p38 and the phosphorylation of p38 (p-p38) in the habenula were assessed. The effect of the antidepressant fluoxetine on depressive-like behaviors, the levels of TNF-α, IL-10, and p38, and the phosphorylation of p38 (p-p38) were investigated. Finally, the effects of SB203580, a p38-specific inhibitor, on depressive-like behaviors and the levels of TNF-α, IL-10, p38, and p-p38 in the habenula were investigated. This study could provide new information for understanding the neuronal mechanisms of depression induced by inflammation.

## Materials and Methods

### Animals

Male Sprague–Dawley rats, weighing 300 ± 20 g, were purchased from Vital River Laboratories (Beijing, China). Rats were housed individually in standard stainless-steel cages, sized 25 cm × 22.5 cm × 30 cm. All rats had free access to food and water. Rats were housed in a temperature- and humidity-controlled room (20∼24°C and 40–70%, respectively) with a fixed 12 h light–dark cycle (07:00-19:00). All rats were acclimated to the housing facilities for 1 week before surgery. Each rat was handled for 3 min daily during the acclimation period to minimize stress responses to experimental manipulations. The experimental procedures were approved by the Institutional Review Board of the Institute of Psychology, Chinese Academy of Sciences and followed the National Institutes of Health Guide for the Care and Use of Laboratory Animals.

### Stereotaxic Surgery

Briefly, after acclimation, the rats were anesthetized by i.p. injection of 1% pentobarbital (35 mg/kg). The heads of the rats were fixed on the stereotaxic apparatus. The scalps were cut after sterilization to expose the bregma and the posterior fontanelle. Stainless-steel guide cannulas were implanted on the lateral ventricles, according to the Chemoarchitectonic Atlas of the Rat Brain (Paxinos and Watson, 2004). The guide cannulas were anchored to the skull with screws and dental cement. Inner cores were inserted into the cannulas in case of blocking.

### Intraventricular Injection

All injections were performed in freely moving animals, as described in our previous studies (Pan et al., 2013; Tang et al., 2016). The microinjector and the inner pipe were connected with a PE pipe, and the inner pipe was inserted into the cannula. LPS was diluted to 100 ng/μl with sterile saline and was infused intracerebroventricularly (i.c.v.), at a dose of 100 ng per rat. This dose was chosen because it has previously been shown to significantly increase immobility and decrease locomotor activity in rats (Pan et al., 2013; Tang et al., 2016, 2017). SB203580 was diluted to 2.5 mg/ml with dimethyl sulfoxide (DMSO) and was infused intracerebroventricularly (i.c.v.), at a dose of 5 μg per rat (Zheng et al., 2004; Zhang et al., 2010). Drug or vehicle was injected by an automatic micropump at a rate of 0.5 μl/min (LPS) or 1 μl/min (SB203580). The microinjector was held still for at least 1 min after the injection to guarantee the sufficient permeation of the drug and to avoid backflow. The cannula was blocked with the inner pipe after injections.

### Experimental Procedures

Experiment 1: The effects of central LPS treatment on depressive-like behaviors, cytokine expression and the phosphorylation of p38 in the habenula

Twenty-eight rats were randomly assigned to two groups (14 rats per group). Rats in the LPS group received central LPS treatment once every 2nd day (i.e., 100 ng/μl on days 1, 3, and 5) three times. This dose was chosen because it has previously been shown to induce significant depressive-like behavior (Tang et al., 2016). The control rats were infused with 1 μl of pyrogen-free physiological saline. Each group was separated into two subgroups: one was used to perform behavioral tests and Western blots, and the other was used to perform immunohistochemistry. After behavioral measurements were taken, rats were decapitated and the habenula were isolated for the determination of cytokine levels and the phosphorylation of p38 by Western blot or immunohistochemistry.

Experiment 2: The effects of fluoxetine on depressive-like behaviors and the altered cytokines and phosphorylation of p38 induced by central LPS treatment

Fifty-six rats were randomly assigned to four groups (14 rats per group). The Flu/LPS groups were administered fluoxetine i.p. at a dose of 10 mg/kg every day for 14 days and LPS treatment once every second day (i.e., 100 ng/μl on days 10, 12, and 14) three times. The fluoxetine dose was chosen because it has previously been shown to significantly alleviate depressive-like behaviors and helplessness in rats (Brenes and Fornaguera, 2009; Hansen et al., 2011; Freund et al., 2013). Each group was separated into two subgroups: one was used to perform behavioral tests and Western blots, and the other was used to perform immunohistochemistry. After behavioral measurements were taken, rats were decapitated and the habenula were isolated to determine the cytokine levels and the phosphorylation of p38 by Western blot or immunohistochemistry.

Experiment 3: The effects of the p38 inhibitor, SB203580, on depressive-like behaviors and the altered cytokines and phosphorylation of p38 induced by central LPS treatment

Forty-eight rats were randomly assigned to four groups (12 rats per group). The SB/LPS groups were administered SB203580 treatment (i.e., 5 μg per rat) for 5 days and LPS treatment every 2nd day (i.e., 100 ng/μl on days 1, 3, and 5) 3 times, 30 min after SB203580 treatment. Each group was separated into two subgroups: one was used to perform behavioral tests and Western blots, and the other was used to perform q-PCR. After behavioral measurements were taken, rats were decapitated and the habenula were isolated to determine cytokine levels and the phosphorylation of p38 by q-PCR or Western blot.

### Behavioral Tests

#### Saccharin Preference Test

The reduced consumption of sweetened solutions by rodents has been used as a measurement for anhedonia, a core symptom of depression. Before the test, rats were deprived of water for 20 h (from 12:00). On the test day, each rat was given two bottles, containing either water or 0.5% saccharin, for 2 h (from 8:00 to 10:00). The amount of each solution consumed was determined by weighing the bottles before and after the 2 h window. The two bottles changed positions every 30 min to avoid the influence of place preference on the experimental results. Rats received unlimited access to water again after the test.

#### Tail Suspension Test

The TST is a standardized test of depressive-like behaviors, in which depression is inferred from an increased duration of immobility. Rats were randomly chosen from each group. Double-deck medical tape (7 cm × 2 cm) was pasted to the middle part of the tail. A small hole was made on the tape to hang the rats on a stainless-steel hook. Each rat was tested for 5 min after a 1-min acclimation period. Immobility was defined as a complete lack of movement in the four limbs and the trunk.

### Quantitative-PCR (q-PCR)

Brains were dissected, and the habenula was isolated using RNase-free instruments and then mechanically homogenized in TRIzol reagent (Invitrogen, MA, United States). RNA was isolated with PureLink^TM^ RNA Mini Kit (Invitrogen, MA, United States). The concentration of RNA was determined using a NanoDrop 2000 spectrophotometric system (Thermo, MA, United States). cDNA was generated using PrimeScript^TM^ RT reagent Kit (TaKaRa, Shiga, Japan), according to the manufacturer’s protocol. cDNA was stored at -20°C until q-PCR reactions were prepared. To detect the mRNA levels of TNF-α and IL-10, q-PCR was performed, employing the primers depicted in **Table 1**. Values were normalized to the expression level of GAPDH, as a housekeeping gene. The PCR reaction was performed on an Stratagene Mx3000P real-time PCR machine (Agilent Technologies, Santa Clara, CA, United States), using SYBR Green mix (Promega, Madison, WI, United States), according to manufacturer’s protocol. A reaction volume of 20 μl per sample was used, consisting of 2 μl cDNA, 0.8 μl each of the corresponding forward and reverse primers, 10 μl SYBR Premix Ex TaqTMII (2×), 0.4 μl ROX Reference Dye (50×) and 6 μl dH_2_O. A two-step PCR was performed with the following cycling program: after an initial activation step (30 s at 95°C), 40 q-PCR cycles were run, composed of 5 s denaturation at 95°C, 31 s annealing at 60°C, and 5 min final extension at 60°C. Reactions were performed in duplicate. Quantification was accomplished by the ΔΔCt-method.

**Table 1:** Sequence of primers for real time polymerase chain reaction.

Gene	Forward primer	Reverse primer
TNF-α	5-AAATGGGCTCCCTCTCATCCAGTTC-3	5-TCTGCTTGGTGGTTTGCTACGAC-3
Il-10	5-CACTGCTATGTTGCCTGCTCT-3	5-TCATTCTTCACCTGATCCACT-3
GAPDH	5-GACATGCCGCCTGGAGAAAC-3	5-AGCCCAGGATGCCCTTTAGT-3

*GAPDH, glyceraldehyde 3-phosphate dehydrogenase; IL, interleukin; TNF, tumor necrosis factor*.

### Western Blot

All rats were executed 24 h after behavioral tests were performed, and the habenula was dissected according to Paxinos & Watson’s rat brain atlas (Paxinos and Watson, 2004). Tissues were lysed by RIPA lysis buffer (pH 7.5, containing 50 mM Tris–HCl, 2 mM EDTA, 2 mM EGTA, 0.05 mM okadaic acid, 1 μM sodium orthovanadate, 5 μg/ml pepstatin A, and 0.5% Nonidet P-40) on ice, then mechanically crushed and homogenized by ultrasonic waves. A BCA protein assay kit (Thermo, MA, United States) was used to measure the total protein concentration in the lysates. Protein was transferred to a 0.2 μm nitrocellulose (NC) filter (Sigma-Aldrich, St. Louis, MO, United States) by electrophoretic transfer after gel separation by 10% sodium dodecyl sulfate polyacrylamide gel electrophoresis (SDS-PAGE). Membranes were blocked by 5% milk overnight at 4°C and washed three times for 10 min with PBST (containing 0.5% Tween-20). Then, the membranes were incubated with primary antibody (Rabbit anti-p38 No: 8690S and rabbit anti-phospho-p38 No: 4511S, 1:1000, Cell Signaling Technology, Danvers, MA, United States. Rat anti-TNF-α No: AF-510-NA and Mouse/Rat anti-IL-10 No: AF519, 0.1 μg/ml, R&D Systems, Minneapolis, MN, United States) and secondary antibody (Goat anti-Rabbit No: ZB-2301 and Goat anti-Mouse No: ZB-2305, 1:10,000, ZSGB-BIO, Beijing, China) for 2 h and 1 h, respectively, followed by ECL and film detection. Membranes were then stripped by stripping buffer and used for the detection of other proteins of interest, as well as GAPDH (mouse anti-GAPDH, No: TA309157, 1:5000, ZSGB-BIO, Beijing, China), as a loading control. The differences in protein expression among different groups were compared by measuring the optical density of the corresponding protein targets using Quantity One 1-D analysis software (UVP, Upland, CA, United States). The relative protein levels were calculated from the ratio of the absorbance of p-p38 or p38 relative to the corresponding GAPDH absorbance for each sample, to correct for small differences in protein loading.

### Immunohistochemistry

Rats were executed by decapitation 24 h after the last saline or LPS injection. Brains were dissected and fixed in 4% paraformaldehyde at room temperature for 48 h. Then, the habenula was dissected and fixed in freshly prepared 4% paraformaldehyde at 4°C for 48 h. Samples were then embedded in paraffin, after dehydration using a graded ethanol series, and cut into pieces at a thickness of 5 μm. The brain slices were dried at 60°C for 1 h; dewaxed twice in xylene, for 15 min each; dehydrated using a graded ethanol series; and washed using deionized water and PBS. Antigen retrieval protocol was as follows: slices were microwaved in citric acid buffer (PH 6.0) for 6 min three times with 2-min intervals, then cooled for 2 h at room temperature (RT); incubated with 3% hydrogen peroxide at room temperature for 30 min; and washed with PBS. Serum blocking was performed with a 5% normal goat serum solution for 1 h at RT. Slices were incubated with 50 μl (15 μg/ml) of primary antibody (Rabbit anti-p38 No: 8690S and rabbit anti-phospho-p38 No: 4511S, 1:1000, Cell Signaling Technology, Danvers, MA, United States. Rat anti-TNF-α No: AF-510-NA and Mouse/Rat anti-IL-10 No: AF519, 0.1 μg/ml, R&D Systems, Minneapolis, MN, United States) at 4°C overnight, using PBS as a negative control and lymph node tissue as a positive control. Slices were incubated with goat two-step method immunochemistry assay kit reagent 1 and reagent 2 for 20 min each at room temperature and washed with PBS. Slices were developed with DAB for 7∼10 min and washed with distilled water. Slices were dehydrated with a gradient alcohol series the samples were mounted on slides. All sections were imaged using a microscope (Leica Microsystems, Wetzlar, Germany). Image Pro-Plus 6.0 was used to analyze the brain areas by calculating the OD values and counting the number of positive cells.

### Statistical Analysis

All data are presented as the mean ± the standard error of the mean (SE). The data were analyzed using Student’s *t*-test or one-way ANOVA, followed by a Tukey’s *post hoc* test where appropriate. The level of significance was set at *p* ≤ 0.05.

## Results

### Effects of Repeated Central Administration of LPS on Depressive-Like Behaviors and the Levels of TNF-α and p-p38 in the Habenula

#### Repeated Central LPS Administration Induced Depressive-Like Behaviors

Student’s *t*-test showed no significant differences in body weight gain between the two groups before LPS administration (Saline group: 6.07 ± 0.82, LPS group: 6.20 ± 0.79). LPS administration significantly reduced the weight of rats compared with the saline group (*p* < 0.001, Saline group: 2.78 ± 1.36, LPS group: -5.63 ± 0.90), indicating that the repeated central administration of LPS negatively affected body weight gain.

**FIGURE 1:**
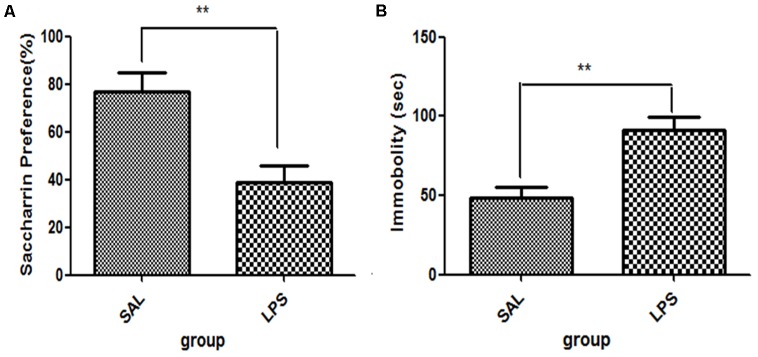
Effects of central LPS administration on depressive-like behaviors. **(A)** Saccharin preference. **(B)** Immobility time in tail suspension test. LPS injection: 100 ng/μl per rat, once every other day for a total of three times. LPS group (*n* = 8), saline group (*n* = 8). Data are presented as the mean ± SE. ^∗∗^*p* < 0.01. LPS: lipopolysaccharide; Sal: saline.

For saccharin preference, *t*-test showed that LPS significantly reduced the saccharin preference scores compared with the saline group (*p* < 0.01, **Figure 1A**), while no significant differences in either total saccharin solution intake or saccharin preference scores were observed between the two groups before LPS administration. These results indicated that the central administration of LPS could induce anhedonia in rats. For immobility time from the tail suspension tests, data showed that the immobility time of rats in the LPS group was significantly longer than that of rats in the saline group (*p* < 0.01, **Figure 1B**).

#### Repeated Central Administration of LPS Increased the Expression of TNF-α in the Habenula

**FIGURE 2:**
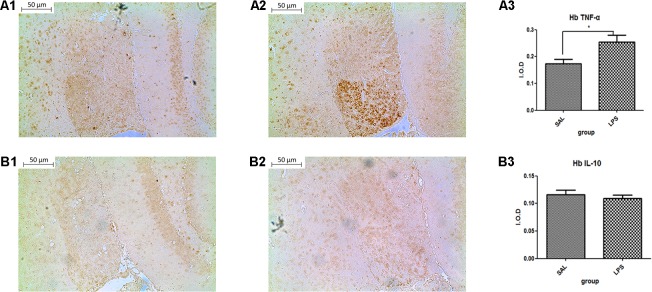
Effects of central saline and LPS administration on TNF-α expression in the habenula (immunohistochemistry). **(A1)** The effects of central saline administration on TNF-α expression in the habenula. **(A2)** The effects of central LPS administration on TNF-α expression in the habenula. **(A3)** Quantized data of the TNF-α positive area after central saline and LPS administration. **(B1)** The effects of central saline administration on IL-10 expression in the habenula. **(B2)** The effects of central LPS administration on IL-10 expression in the habenula. **(B3)** Quantized data of the IL-10 positive area after central saline and LPS administration. LPS injection: 100 ng/μl per rat, once every other day for a total of three times. LPS group (*n* = 6), saline group (*n* = 6). Data are presented as the mean ± SE. ^∗^*p* < 0.05. LPS, lipopolysaccharide; Sal, saline.

Data from the immunohistochemistry analysis showed that the expression of TNF-α in the habenula of the LPS group was significantly higher than in the saline group (*p* < 0.05) after LPS administration (**Figure 2**). There was no significant difference observed in the expression of IL-10 between the LPS and saline groups.

#### Repeated Central Administration of LPS Stimulated the Phosphorylation of p38 in the Habenula

**FIGURE 3:**
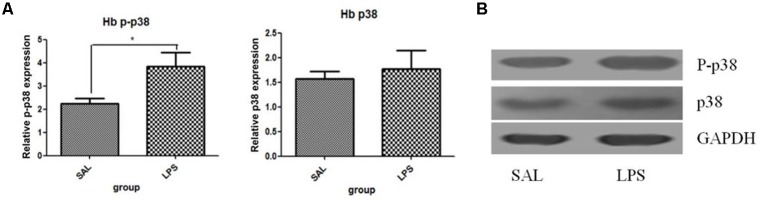
Effects of repeated central LPS administration on p38 phosphorylation in the habenula (Western blot). **(A)** Quantized data of the effect of LPS on p38 phosphorylation in the habenula. **(B)** Representative Western blot of p-p38 and p38 in the habenula. LPS injection: 100 ng/μl per rat, once every other day,on days 1, 3, and 5, for a total of three times. Veh/Sal (*n* = 6), Veh/LPS (*n* = 6), and SB/Sal (*n* = 6). Data are presented as the mean ± SE. ^∗^*p* < 0.05. LPS, lipopolysaccharide; Sal, saline.

**FIGURE 4:**
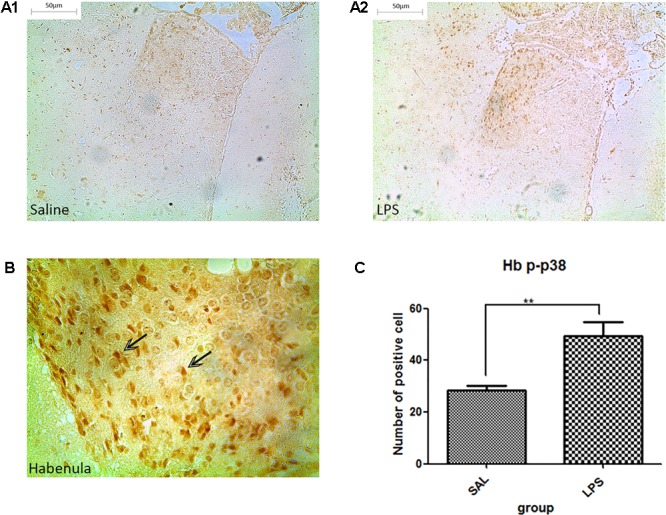
Effects of repeated central LPS administration on p38 phosphorylation in the habenula (Immunohistochemistry). **(A1)** Effects of saline administration on p38 phosphorylation in the habenula. **(A2)** Effects of repeated central LPS administration on p38 phosphorylation in the habenula. **(B)** Positive cells containing p-p38 in the habenula after repeated LPS administration. **(C)** Quantized data of the number of p-p38 positive cells after LPS administration in the habenula. LPS injection: 100 ng/μl per rat, once every other day, on days 1, 3, and 5, for a total of three times. Saline group (*n* = 6), LPS group (*n* = 6). Data are presented as the mean ± SE. ^∗∗^*p* < 0.01. LPS: lipopolysaccharide; Sal: saline.

Western blot analysis showed that the protein level of p-p38 in the habenula of the LPS group was much higher than that of the saline group (*p* < 0.01, **Figure 3**), while the total protein level of p38 was not significantly different between the two groups. Immunohistochemistry analysis also demonstrated that the number of p-p38 positive cells in the habenula of the LPS group was increased compared to the saline group (*p* < 0.01, **Figures 4A1,A2,C**). Morphology analysis indicated that the p-p38-positive cells were primarily microglial cells located in the habenula (**Figure 4B**).

### Fluoxetine Reversed the Depression-Like Behaviors and the Changes in Cytokine Expression and the Phosphorylation of p38 MAPK Induced by LPS

#### Fluoxetine Significantly Reversed the Depression-Like Behaviors Induced by LPS

For body weight gain, a one-way ANOVA analysis showed that the body weight gained by rats in the Veh/LPS group and the Flu/Sal group was significantly lower than that of rats in the Veh/Sal group (*p* < 0.001). There was no significant difference between the Flu/LPS group and the Veh/LPS group in body weight gain. These data indicated that both fluoxetine and LPS decreased the weight gain of rats, but the effect of the combination of the two drugs on body weight did not differ from LPS treatment alone.

**FIGURE 5:**
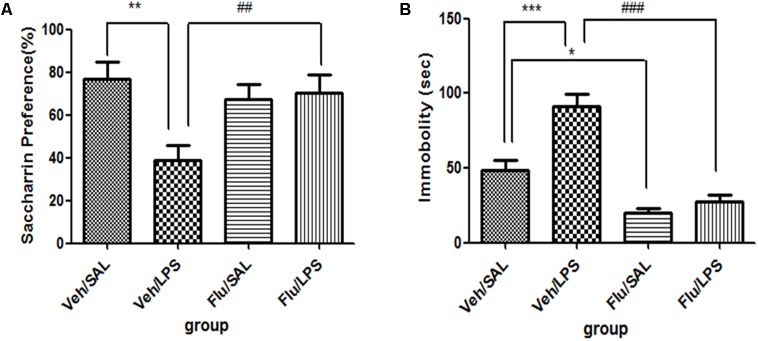
Effects of fluoxetine on depressive-like behaviors induced by LPS. **(A)** Saccharin preference. **(B)** Immobility time in tail suspension test. Fluoxetine injection: i.p. 10 mg/kg per rat every day for a total of 14 days, LPS injection: 100 ng/μl per rat, once every other day, on days 10, 12, and 14, for a total of three times. Veh/Sal (*n* = 8), Veh/LPS (*n* = 8), Flu/Sal (*n* = 8), and Flu/LPS (*n* = 8). Data are presented as the mean ± SE. ^∗^*p* < 0.05, ^∗∗^*p* < 0.01, ^∗∗∗^*p* < 0.001, ^##^*p* < 0.01, ^###^*p* < 0.001. Flu, fluoxetine; LPS, lipopolysaccharide; Sal, saline.

For saccharin preference, a one-way ANOVA showed a significant difference in the saccharin preference score of rats among different groups after LPS and fluoxetine treatment [*F*(3,28) = 4.782, *p* < 0.01]. *Post hoc* analyses revealed that the saccharin preference in the Veh/LPS group was significantly lower in the Veh/Sal group after treatment (*p* < 0.01). The saccharin preference score of rats in the Flu/LPS group was much higher than that of rats in the Veh/LPS group (*p* < 0.01, **Figure 5A**). These data indicated that fluoxetine could significantly reverse the anhedonia induced by LPS.

For immobility time in the tail suspension tests, a one-way ANOVA showed significant differences in the immobility time of rats among different groups after LPS and fluoxetine treatment [*F*(3,28) = 28.387, *p* < 0.001]. *Post hoc* analyses revealed that the immobility time of rats in the Veh/LPS group was much longer than that of rats in the Veh/Sal (*p* < 0.001, Fig. 5B). The immobility time of rats in the Flu/Sal group was significantly decreased compared to rats in the Sal/LPS group (*p* < 0.001, **Figure 5B**). The immobility time of rats in the Flu/LPS group was significantly decreased compared to rats in the Veh/LPS group (*p* < 0.001, **Figure 5B**). The data indicated that fluoxetine could significantly alleviate despair behaviors induced by LPS.

Taken together, these data suggest that Fluoxetine treatment significantly reversed the depression-like behaviors induced by LPS.

#### Fluoxetine Significantly Reversed the Altered Expression of Cytokines Induced by LPS

The expression of TNF-α and IL-10 showed significant differences among the four groups [one-way ANOVA, TNF-α, *F*(3,20) = 6.508, *p* < 0.01; IL-10, *F*(3,20) = 7.261, *p* < 0.01].

**FIGURE 6:**
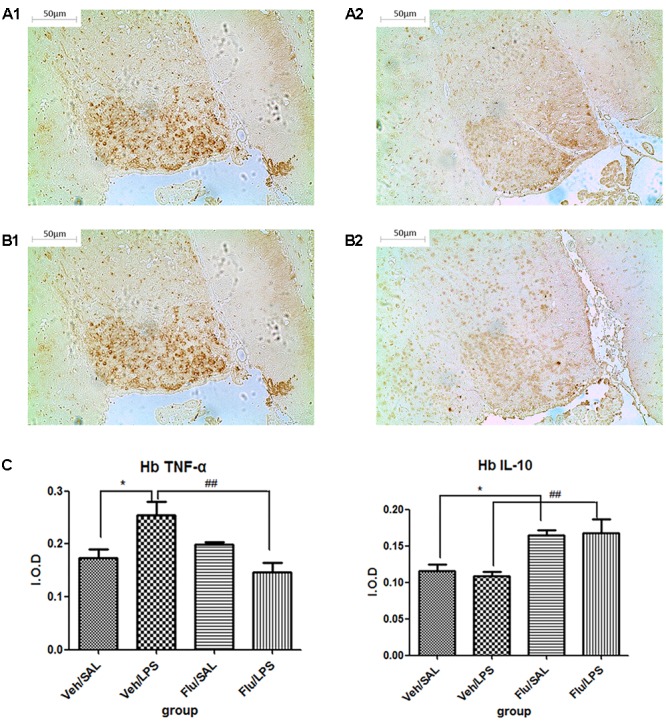
Effects of fluoxetine on TNF-α and IL-10 expression in rats under neuroinflammation conditions (immunohistochemistry). **(A1)** TNF-α expression in the habenula of the Veh/LPS group. **(A2)** TNF-α expression in the habenula of the Flu/LPS group. **(B1)** IL-10 expression in the habenula of the Veh/LPS group. **(B2)** IL-10 expression in the habenula of the Flu/LPS group. **(C)** Quantized data of the effects of fluoxetine and LPS on TNF-α and IL-10 expression in the habenula. Fluoxetine injection: i.p. 10 mg/kg per rat every day for a total of 14 days, LPS injection: 100 ng/μl per rat, once every other day, on days 10, 12, and 14, for a total of three times. Veh/Sal (*n* = 6), Veh/LPS (*n* = 6), Flu/Sal (*n* = 6), and Flu/LPS (*n* = 6). Data are presented as the mean ± SE. ^∗^p < 0.05, ^##^p < 0.01. Flu, fluoxetine; LPS, lipopolysaccharide; Sal, saline.

Further *post hoc* analyses indicated that the expression of TNF-α in the Veh/LPS group was significantly increased compared with the Veh/Sal group (*p* < 0.05, **Figure 6A1**); fluoxetine treatment significantly decreased the increased expression of TNF-α in the habenula (*p* < 0.01, **Figure 6A2**). Fluoxetine also increased the expression of IL-10 in the habenula in both the Flu/Sal group (*p* < 0.05, **Figure 6B1**) and the Flu/LPS group (*p* < 0.01, **Figure 6B2**), indicating the anti-depressive effect of fluoxetine may be the result of both decreasing the expression of TNF-α and increasing the expression of the anti-inflammatory cytokine IL-10.

#### Fluoxetine Significantly Reversed the Altered Phosphorylation of p38 Induced by LPS

**FIGURE 7:**
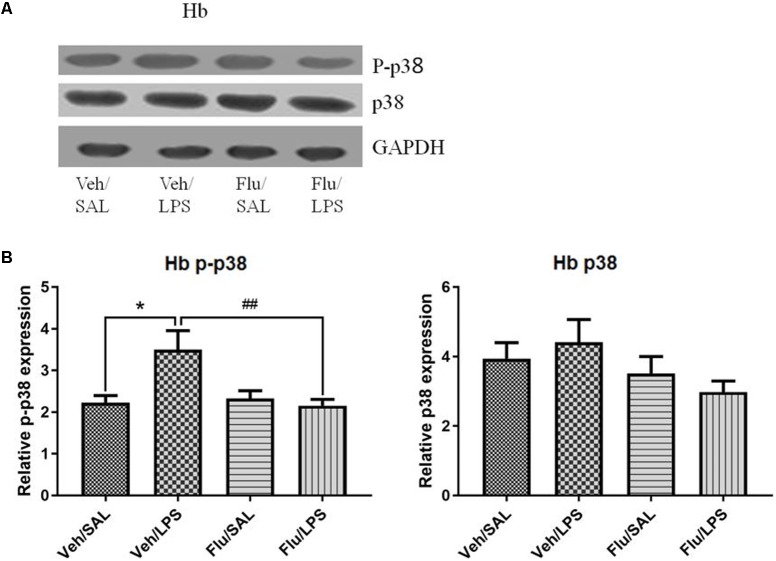
Effects of fluoxetine on p38 phosphorylation in the habenula in rats under neuroinflammation conditions (Western blot). **(A)** Representative Western blot for the effects of fluoxetine on p38 phosphorylation in the habenula under neuroinflammation conditions. **(B)** Quantized data of the effects of fluoxetine and LPS on p-p38 and p38 in the habenula. Fluoxetine injection: i.p. 10 mg/kg/ rat every day, for a total of 14 days, LPS injection: 100 ng/μl per rat, once every other day, on days 10, 12, and 14, for a total of three times. Veh/Sal (*n* = 8), Veh/LPS (*n* = 8), Flu/Sal (*n* = 8), and Flu/LPS (*n* = 8). Data are presented as the mean ± SE. ^∗^*p* < 0.05, ^##^*p* < 0.01. Flu, fluoxetine; LPS, lipopolysaccharide; Sal, saline. I.O.D.

One-way ANOVA analysis showed significant differences in the phosphorylation of p38 among the treatment groups [*F*(3,20) = 6.104, *p* < 0.01]. *Post hoc* analyses revealed that LPS significantly increased the phosphorylation of p38 in the habenula (*p* < 0.05, **Figure 7**), which was inhibited by fluoxetine, without affecting the total protein level of p38 (*p* < 0.01, **Figure 7**), indicating that fluoxetine may work by inhibiting the p38 MAPK signaling pathway in the habenula.

### Blocking the p38 Pathway Reversed Depressive-Like Behaviors Induced by LPS and Reduced the Activation of Pro-inflammatory Cytokines and the Phosphorylation of p38 in the Habenula

#### SB203580 Significantly Reversed the Depressive-Like Behaviors Induced by LPS Administration

For body weight gain, a one-way ANOVA analysis indicated that the weight gain of rats was significantly different among the treatment groups after LPS and SB203580 treatments [*F*(3,20) = 169.151, *p* < 0.001]. *Post hoc* analyses revealed that the weight gain of rats in the Veh/LPS group was significantly less than that of rats in the Veh/Sal group (*p* < 0.001). SB203580 did not show a reversing effect on weight reduction induced by LPS. SB203580 had no effect on weight gain.

**FIGURE 8:**
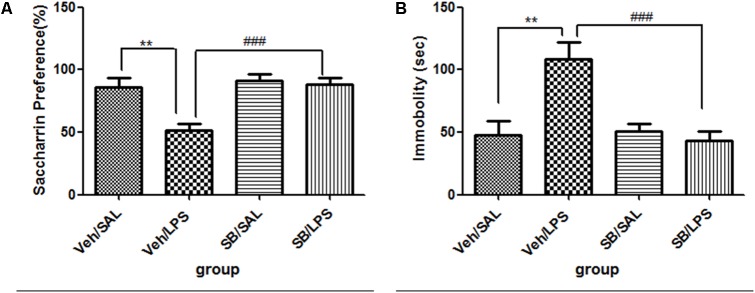
Effects of SB203580 on depressive-like behaviors induced by LPS. **(A)** Saccharin preference. **(B)** Immobility time in tail suspension test. SB203580 injection: 5 μg per rat every day, for a total of 5 days, LPS injection: 100 ng/μl per rat, once every other day, on days 1, 3, and 5, for a total of three times. Veh/Sal (*n* = 6), Veh/LPS (*n* = 6), SB/Sal (*n* = 6), and SB/LPS (*n* = 6). Data are presented as the mean ± SE. ^∗∗^*p* < 0.01, ^###^*p* < 0.001. SB, SB203580; LPS, lipopolysaccharide; Sal, saline.

For saccharin preference, a one-way ANOVA analysis indicated a significant difference in the saccharin preference of rats among treatment groups after LPS and SB203580 treatment [*F*(3,20) = 6.104, *p* < 0.01]. *Post hoc* analyses revealed that the saccharin intake of the Veh/LPS group was significantly reduced when compared with the Veh/Sal group (*p* < 0.01, **Figure 8A**). The saccharin intake of the SB/LPS group was increased compared to that of the Veh/LPS group (*p* < 0.001, **Figure 8A**). The results indicated that SB203580 reversed the anhedonia induced by LPS in rats. For the tail suspension test, a one-way ANOVA showed significant differences in the immobility time of rats among different groups after LPS and SB203580 treatment. *Post hoc* LSD analyses revealed that the immobility time of rats in the Veh/LPS group was much longer than for rats in the Veh/Sal group (*p* < 0.01, **Figure 8B**). The immobility time of rats in the SB/LPS group was significantly decreased when compared to rats in the Sal/LPS group (*p* < 0.001, **Figure 8B**). These data indicated that SB203580 alleviated immobility behavior induced by LPS.

#### SB203580 Significantly Reversed the Expression of Cytokines in the Habenula That Was Induced by LPS Administration

**FIGURE 9:**
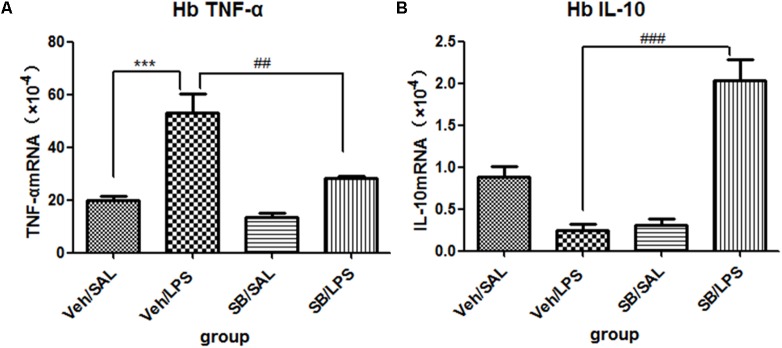
Effects of SB203580 on TNF-α and IL-10 expression levels in rats under neuroinflammation conditions (Q-PCR). **(A)** The effects of SB203580 on TNF-α expression in the habenula induced by central LPS administration. **(B)** The effects of SB203580 on IL-10 expression in the habenula induced by central LPS administration. SB203580 injection: 5 μg per rat every day, for a total of 5 days, LPS injection: 100 ng/μl per rat, once every other day, on days 1, 3, and 5, for a total of three times. Veh/Sal (*n* = 6), Veh/LPS (*n* = 6), SB/Sal (*n* = 6), and SB/LPS (*n* = 6). Data are presented as the mean ± SE. ^∗∗∗^*p* < 0.001, ^##^*p* < 0.01, ^###^*p* < 0.001. SB, SB203580; LPS, lipopolysaccharide; Sal, saline.

A one-way ANOVA analysis indicated that LPS and SB203580 treatments resulted in significant difference in the mRNA levels of TNF-α [*F*(3,20) = 19.28, *p* < 0.001] and IL-10 [*F*(3,20) = 30.66, *p* < 0.001] among different treatment groups. *Post hoc* analyses revealed that repeated central LPS administration significantly increased the mRNA levels of TNF-α (*p* < 0.001, **Figure 9A**) and decreased the mRNA levels of IL-10 (**Figure 9B**). SB203580 treatment reversed the increase in TNF-α mRNA levels (*p* < 0.01, **Figure 9A**) induced by LPS and significantly increased the IL-10 mRNA levels (*p* < 0.001, **Figure 9B**) in the habenula.

#### SB203580 Significantly Reversed the Altered Phosphorylation Levels of p38 in the Habenula That Were Induced by LPS

**FIGURE 10:**
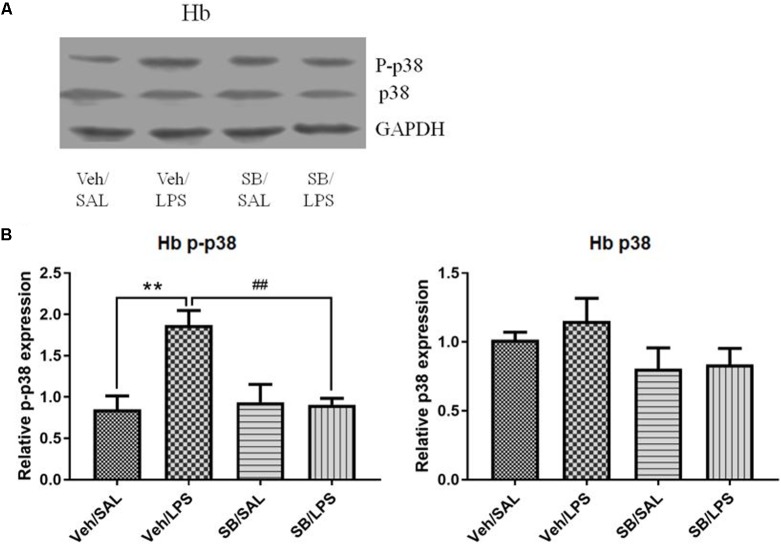
Effects of SB203580 on p38 phosphorylation in the habenula in rats under neuroinflammation conditions (Western blot). **(A)** Representative Western blot for the effects of SB203580 on p-p38 and p38 in the habenula. **(B)** Quantized data of the effects of SB203580 and LPS on p38 phosphorylation and p38 levels in the habenula. SB203580 injection: 5 μg per rat every day, for a total of 5 days, LPS injection: 100 ng/μl per rat, once every other day, on days 1, 3, and 5, for a total of three times. Veh/Sal (*n* = 6), Veh/LPS (*n* = 6), SB/Sal (*n* = 6), and SB/LPS (*n* = 6). Data are presented as the mean ± SE. ^∗∗^*p* < 0.01, ^##^*p* < 0.01. SB, SB203580; LPS, lipopolysaccharide; Sal, saline.

A one-way ANOVA analysis indicated that LPS and SB203580 treatments resulted in significant differences in the phosphorylation levels of p38 among different treatment groups [*F*(3,20) = 8.693, *p* < 0.001]. *Post hoc* analyses revealed that SB203580 treatment significantly decreased the phosphorylation levels of p-p38 that were induced by LPS in the habenula when compared to the Veh/LPS group (*p* < 0.01, **Figure 10**), while the total protein level of p38 was not significantly different across the groups.

## Discussion

The present study revealed that neuroinflammation induced depressive-like behaviors, which were accompanied by increased levels of TNF-α and p-p38 in the habenula. Both the p38 inhibitor SB203580 and fluoxetine normalized the increased expression of TNF-α that was induced by LPS and up-regulated the levels of the anti-inflammatory cytokine IL-10 in the habenula. Both the p38 inhibitor SB203580 and fluoxetine also normalized the changes in p38 phosphorylation and reversed the depressive-like behaviors. These findings show, for the first time, that the habenula is involved in the pathophysiology of behavioral depression induced by neuroinflammation and that the p38 pathway plays an important role in mediating inflammation-related depression.

Lipopolysaccharide has been used as a stimulator for depressive-like behaviors in animals and humans for many years (Russo et al., 2014; Adzic et al., 2015; Mitic et al., 2015). Our previous study showed that triple central LPS administration could induce depressive-like behaviors in rats, which was accompanied by the increased expression of pro-inflammatory cytokines in the hippocampi of rats (Tang et al., 2016). The present study found that LPS could induce depressive-like behaviors and a pro-inflammatory response, namely, the up-regulation of TNF-α, in the habenula. The pro-inflammatory response is in concordance with previous work, in which both acute and chronic restraint stress could induce the expression of IL-18 in the habenula (Sugama et al., 2002).

Although the hippocampus was considered to be the most important area involved in depression, evidence suggests that the habenula plays a very important role in the pathogenesis of depression (Li et al., 2016; Lawson et al., 2017; Seo et al., 2017). Most studies of depression have been performed on the LHb (Li et al., 2016; Seo et al., 2017), although some studies have shown that the MHb was also involved in depression. One study reported that the volume of the MHb, along with the LHb, was decreased in patients diagnosed with major depressive disorder (MDD) (Ranft et al., 2010). A recent study indicated that mice with dorsal MHb lesions showed a significant reduction in sucrose solution preference when compared with control mice, indicating that the MHb participates in the anhedonia symptom of depression (Hsu et al., 2014). The MHb is the region where mast cells encounter the blood–brain barrier, and the unique assembly of mast cells in the MHb implicates an important immune-sensitive brain region (Silver et al., 1996). However, there have been few studies linking immune activation in the habenula to depression. Our present study, for the first time, found that LPS could significantly increase the expression of TNF-α in the habenula, indicating that the habenula is sensitive to immune challenges. We further found that the decreased expression of TNF-α in the habenula was associated with the alleviation of LPS-induced depressive-like behaviors, suggesting that TNF-α in the habenula may participate in LPS-induced depressive-like behaviors.

Regarding the effects of MAPK on depression, our previous study showed that central LPS administration could induce depressive-like behaviors and decrease the phosphorylation level of the MAPK ERK in the hippocampi of rats. Exogenous FGF2 infusions prevented the decrease in ERK1/2 phosphorylation and reversed the depressive-like behaviors induced by neuroinflammation (Tang et al., 2017). Inhibiting the expression of p-ERK could directly induce depressive-like behaviors (Qi et al., 2009). However, the present study found that p38 MAPK showed opposite properties to those reported for ERK. Our data showed that LPS induced the activation of P38, which was associated with the occurrence of depressive-like behaviors. The direct down-regulation of p-p38 by the inhibiter SB203580 could significantly alleviate depressive symptoms induced by LPS, suggesting that P38 activation in the habenula is also a key process in the induction of depressive-like behaviors.

Although the p38 MAPK pathway plays a central role in regulating a wide range of inflammatory responses in many different cells, including microglia (Lee et al., 1994; Birrell et al., 2000; Haddad et al., 2001; Li et al., 2015; Cakmak et al., 2017), p38 MAPK as a mediator in neuroinflammation-induced depression has not been explored. Our present study showed that inflammation activated P38 MAPK, and the SB203580 inhibitor blocked the P38 activation and the subsequent increase in TNF-α, at the same time that depressive-like behaviors were reversed. More importantly, we found that SB203580 increased the levels of the anti-inflammatory cytokine IL-10 in the habenula. All these results indicate that P38 MAPK is not only the molecular target for the biosynthesis of TNF-α but is also a target for anti-inflammatory and anti-depression drug therapy.

In accordance with the above results, the present work also demonstrated that fluoxetine showed a similar reversing effect as SB203580 on the behavioral and molecular changes. It should be noted that, although an *in vitro* study has shown that fluoxetine could decrease the level of TNF-α by inhibiting the p38 signaling pathway (Liu et al., 2011), there has been a lack of *in vivo* evidence to confirm this finding. Our work indicated that fluoxetine could not only down-regulate the levels of p38 and TNF-α but could also increase the levels of the anti-inflammatory cytokine IL-10 in the habenula, as well as reverse the depressive-like behaviors. Fluoxetine is a widely used antidepressant, which has been thought to act by inhibiting 5-hydroxytryptamine (5-HT) reuptake in the central nervous system (Cipriani et al., 2005). Our discovery adds new information for understanding the pharmacological mechanism of the antidepressant action of fluoxetine and further provides new evidence supporting the assumption that the p38 signaling pathway may participate in the pathogenesis of depression by modulating the inflammatory environment in the habenula.

The finding that the P38 signaling pathway affects depressive symptoms may also be interpreted in other ways. The first interpretation involves the regulation of monoamine systems, as the habenula is one of few regions that influence both the dopamine and serotonin system (Luo et al., 2015). P38 activation can stimulate the expression of the serotonin transporter (SERT), the neuronal 5-HT transporter, which is used as a major pharmacological target for depression treatment. SERT is the target of effective antidepressant drugs, and its gene variants have been implicated as risk factors for MDD (Owens and Nemeroff, 1994). Studies have shown that the p38 pathway is an important pathway mediating SERT activation induced by endotoxins in mice (Baganz et al., 2015). SERT activation is paralleled with increasing immobility time in FST and TST induced by LPS, which can be reversed by the pretreatment of SB203580 (Zhu et al., 2010). The second possible interpretation could involve the glucocorticoid receptor (GR). Hyperactivity of the HPA axis is a common feature of patients with major depression, and one reason might be the lack of negative feedback effects from glucocorticoids (Pariante and Miller, 2001). By combining with the GR, glucocorticoids exert a feedback inhibition effect on numerous pathways (Pariante and Miller, 2001; Tsigos and Chrousos, 2002). Previous studies have found that the p38 MAPK signaling pathway may be a key pathway mediating the activation and normal function of glucocorticoids by mediating the expression and function of the GR (Wang et al., 2004). The activation of the p38 signaling pathway could inhibit the normal translocation of the GR from the cytoplasm to the nucleus, which could be a contributor to the physiology of major depression (Wang et al., 2004). In addition, p38 signaling could increase the production of pro-inflammatory cytokines, such as TNF-α (Lee et al., 1994), which can directly inhibit GR function and cause glucocorticoid resistance (Miller et al., 1999; Pace et al., 2007). SB203580 could block p38 activation and alleviate glucocorticoid resistance, contributing to the recovery of normal GR function and to the recovery of depression. The third possible interpretation could involve the modulation of brain derived neurotrophic factor (BDNF). Research indicates that chronic treatment with antidepressants affects several proteins related to neuroplasticity, particularly BDNF (Alboni et al., 2010). Multiple lines of evidence suggest that BDNF is integral to both the pathophysiology of depression and the therapeutic mechanisms of antidepressants (Zhang et al., 2016). It has been reported that BDNF has an attenuating effect on the phosphorylation of p38 MAPK in primary cell cortical cultures (Criscuolo et al., 2015). Decreased BDNF levels in the hippocampal and cortical tissues were also found in LPS-treated animals (Alboni et al., 2010). Increased BDNF levels have been shown to promote neuronal plasticity, which is beneficial for the alleviation of depressive-like behaviors in depressed patients (Cai et al., 2015). Concerning the BDNF neuroprotective effects, it is possible that antidepressants that inhibit P38 in inflammation-associated depression may act by promoting BDNF cellular processes linked to neuroplasticity. Above all, the etiology of depression is complicated, and multiple factors have been shown to be involved in the pathology of this disease (Ferrari and Villa, 2017). From the wide spectrum of neuromodulation factors and cytokines, it appears that the p38 MAPK pathway may be involved in depressive-like behaviors in sophisticated and interactive ways. The interactions among various neuromodulation factors, neurotransmitters, and cytokines require further investigation.

In conclusion, central LPS administration induced depressive-like behaviors, increased the levels of p38 phosphorylation in microglial cells, and increased the expression of TNF-α in the habenula. Fluoxetine reversed the depressive-like behaviors, normalized the levels of p38 phosphorylation and the pro-inflammatory cytokine TNF-α and increased the levels of the anti-inflammatory cytokine IL-10. Furthermore, SB203580, a p38-specific inhibitor, could also reverse the LPS-induced depressive-like behaviors, as well as the changes in the TNF-α and IL-10 expression levels in the habenula, by blocking the p38 MAPK pathway. The present findings suggest that the habenula is involved in the pathophysiology of behavioral depression induced by neuroinflammation and that the p38 pathway may serve as a novel mechanism-based target for the treatment of inflammation-related depression.

## Author Contributions

W-jL supervised the overall research. Y-qP performed the experiments and collected the data. Y-qP, Y-wZ, and M-mT analyzed and interpreted the data. Y-wZ and W-jL wrote and revised the manuscript.

## Conflict of Interest Statement

The authors declare that the research was conducted in the absence of any commercial or financial relationships that could be construed as a potential conflict of interest. The reviewer CM and handling Editor declared their shared affiliation.
